# Home delivery and associated factors among women who gave birth after antenatal care follow-up in the last 6 months in Jabitehnan District, Northwest Ethiopia: mixed community-based study

**DOI:** 10.3389/fgwh.2024.1458453

**Published:** 2024-12-06

**Authors:** Bewket Yeserah Aynalem, Tamene Zerihun, Addisu Alehegn Alemu, Belsity Temesgen Meselu, Liknaw Bewket Zeleke, Getachew Mullu Kassa

**Affiliations:** ^1^ Department of Midwifery, Debre Markos University, Debre Markos, Ethiopia; ^2^ School of Women’s and Children’s Health, University of New South Wales Sydney, Sydney, NSW, Australia

**Keywords:** home delivery, magnitude, Jabitehnan, District, Ethiopia

## Abstract

**Introduction:**

Maternal mortality is a global issue, with developing countries accounting for over 99% of maternal deaths, with 30% of Ethiopian mothers dying from pregnancy-related causes. This study aimed to assess the magnitude, associated factors, and reasons for home delivery among women who gave birth after antenatal care follow-up in the last 6 months in Jabitehnan District, Northwest Ethiopia.

**Methods:**

A mixed community-based study was conducted on women who gave birth after antenatal care follow-up in the last 6 months. Data was collected through face-to-face interviews and structured questionnaires. Bivariate and multivariable logistic regressions were performed to identify factors associated with home delivery. Qualitative data were collected through focus group discussions and in-depth interviews and analyzed using a thematic content analysis method.

**Result:**

In this study, the magnitude of home delivery after ANC follow-up among mothers who gave birth in the last 6 months was 37%, with a 95% CI of 32.5 and 41.5. Pregnancy plan [AOR: 4.56 (2.65, 7.86)], experience of abortion AOR: 3.01 [1.631, (5.55)], ANC follow-up at public hospitals [AOR: 1.89 (1.119, 3.18)], and pregnant mothers visited at home by healthcare providers during their ANC follow-up absent [AOR: 1.61 (1.02, 2.53)] were predictors of home delivery. Poor counseling during ANC, and lack of pregnancy plans, traditions, and beliefs were reasons for home delivery.

**Conclusion:**

The study reveals a high magnitude of home delivery as compared to a study done in Bahir Dar (21.2%). Factors include pregnancy plans, health institution type, abortion experience, and absence from antenatal care. Poor counseling during ANC, and lack of pregnancy plans, traditions, and beliefs were reasons for home delivery. Health professionals should receive training in communication and counseling techniques, and they should encourage mothers to plan their pregnancies and visit facility delivery services during ANC follow-up.

## Introduction

Maternal mortality and morbidity are still tragedies for developing countries. Nowadays, around 1,000 maternal deaths happen every day, and more than 99% of maternal mortality occurs in low-income countries (1, 2). The most common risk factors for this tragedy are infection, hemorrhage, obstructed labor, abortion, and hypertension in pregnancy (3). From this, 75% of maternal mortalities are due to direct obstetric causes, which are common in Africa (4, 5).

In developing countries, the majority of maternal mortalities occur in Sub-Saharan Africa (SSA), and the majority are child-bearing women who are in danger of death during labor due to pregnancy-related causes (6). In Ethiopia, slightly over one in 4 live births in the five years preceding the survey were delivered by a skilled provider (28%) or in a health facility (26%). The percentage of live births delivered by a skilled provider remained virtually unchanged for 5 years after 2000, but increased substantially after 2005; from 6% in the 2000 and 2005 EDHS to 10% in the 2011 EDHS, and reached 28% in the 2016 EDHS. A similar trend is observed for the percentage of live births that occurred in a health facility; it increased from 5% in the 2000 and 2005 EDHS surveys, to 10% in the 2011 EDHS, and to 26% in the 2016 EDHS (7).

The percentage of deliveries in a health facility is more than twice from 10% the 2011 EDHS; while home delivery decreased slightly from 90% to the current level of 64%. Even though ANC follow-up and institutional delivery have significant associations, of women attending antenatal care visits, 36% of them end up with home delivery (7).

According to Jabitehnan District Health Office 2018 annual report, has coverage of ANC 87% with low health facility delivery of 54%. Many factors affect the place of delivery like lack of information and adequate knowledge about danger signs during pregnancy and labor, quality of services, site service utilized, and previous experience (8).

Trends and the rates of maternal mortality and level of home delivery and its associated factors are essential for different interventions, resource mobilization, planning, and evaluation of progress towards sustainable development goal 3 (9).

The Federal Ministry of Health/FMOH in Ethiopia has applied multi-pronged approaches to reducing maternal and newborn morbidity and mortality. Improving access and strengthening facility-based maternal services is one such approach, and is also a health sector development plan/HSDP strategic objective (10).

Despite these activities, maternal mortality and morbidity are still high, and they influence the social, political, and economic aspects of the country (2). Even though the problem is high, maternal mortality and morbidity could be lowered by creating different methods, like increasing the quality and number of skilled birth attendants, and by making the health institution more convenient by fulfilling the necessary materials needed for quality delivery services (11). Therefore, to minimize maternal mortality and morbidity, the place of delivery and a safe environment have a significant role in avoiding maternal and neonatal mortality, which is imposed by an unhygienic environment and other technical problems (12).

Therefore, the purpose of this study was to assess the magnitude, associated factors, and reasons for home delivery among women who gave birth after antenatal care follow-up in the last 6 months in Jabitehnan District, Northwest Ethiopia.

## Methods

### Study area and period

The study was conducted in Amhara regional state, West Gojjam Zone, and Jabitehnan district from September 1 to 30, 2019. This area is surrounding Fenote-Selam Town, located 387 KM and 178 KM from Addis Ababa and Bahir Dar, respectively. It has an estimated area of 1,169.5 km^2^. The district has 38 kebeles and an estimated total population of 225,558 in 2019. Of this, 113,907 (50.5%) are females. 26,859 (23.58%) are reproductive-age women. The district has 11 health centers, 38 health posts, and six private clinics that provide different maternity and child health services.

### Study design

Community-based qualitative and quantitative study methods were employed.

### Study participants

The source population was all women who gave birth within the last 6 months of ANC follow-up in the Jabitehnan district. The study population was all women who gave birth within the last 6 months after ANC follow-up in the selected kebeles of the district.

### Eligibility criteria

All women who had ANC follow-ups and gave birth in the Jabitehnan district were included in the study. However, all women who were not residents of the Jabitehnan district and all women who were critically ill were excluded from this study.

### Sample size determination

The sample size was determined by the single population proportion formula (13). The expected proportion of home delivery (75.3%) from the previous study in the Gozamen district (9) and a 5% confidence limit (margin of error) were used.

$$\begin{array}{rl} \mathrm{initial}\;\mathrm{sample}\;\mathrm{size} & ={\left(Z\frac{a}{2}\right)}^{2}\;\times \;\frac{\;p(1-p)}{w2}=(1.96{)}^{2}\;\\ & \;\times \;\frac{0.753(1-0.753)}{0.05^{2}}=286 \end{array}$$

Considering a 1.5 design effect and a 10% non-response rate (286 × 1.5 × 0.1), the final sample size was 472.

In the qualitative study, a total of six to eight individuals were conducted with mothers who had home deliveries after their ANC follow-up. A total of 24 women who gave birth at home with three sessions participated in the focused group discussions (FGD) and six women participated in in-depth interviews (IDIs).

### Sampling technique

**Figure 1:**
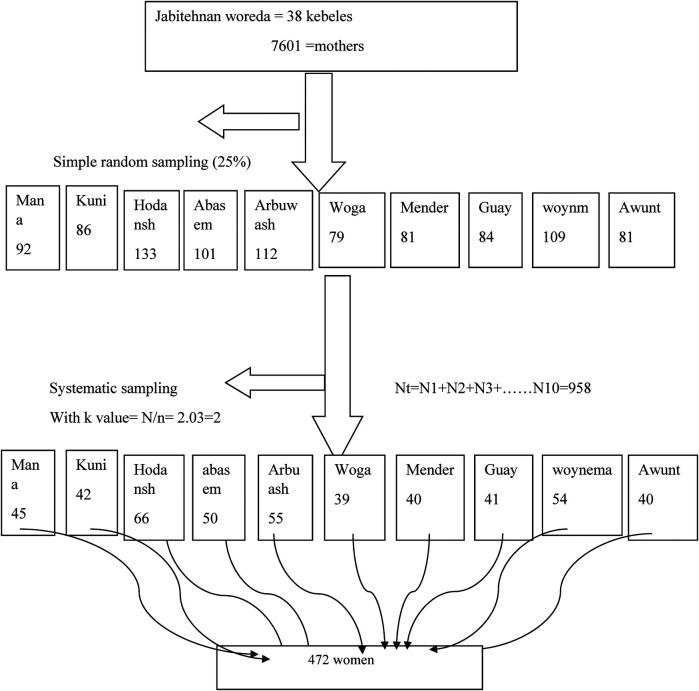
Schematic presentation of the sampling procedure for prevalence and associated factors of home delivery among women who gave birth after antenatal care in the last 6 months of Jabitehnan District, 2019.

The multistage sampling technique was carried out. First, 25% of the 38 kebeles that are found in the study area were randomly selected with the lottery method, and then study participants were allocated proportionally to each randomly selected kebele of the district to determine the total sample size of the study. Participants were selected using systematic sampling techniques from randomly selected kebele at every second interval (Figure 1).

A purposive sampling technique was employed to select qualitative study participants. The selection was based on gravid status, age, and educational status. These participants were selected in six kebeles.

### Study variables

**Dependent variable:** home delivery.

#### Independent variables

**Socio-demographic factors:** age of mother, marital status, religion, education, occupation, ethnicity, income.

**Obstetric history:** previous obstetric history, gravidity, parity, the experience of prolonged labor place of the previous delivery, pregnancy plan, place where ANC attends, number of ANC visits, the experience of stillbirth.

**Health service-related:** time to reach a health facility, media exposure, counseling and communication with health providers, husbands, traditional birth attendants (TBAs), and family influence about the place of delivery preference.

**Health service experience**: the place of current ANC service, place of recent delivery, time to get ANC, the experience of abortion, privacy protection, and knowledge about the free charge of the service.

**Knowledge of obstetric danger signs:** excessive vaginal bleeding, blurred vision, convulsion, and high-grade fever.

**Discussion habit and maternal decision power on a place of delivery:** choice of place of delivery.

**Cultural and traditional factors:** birth assistant.

#### Operational definitions

**Home delivery:** delivery that is not taken place at health facilities (hospitals, health centers, and any private health facilities) (9).

**ANC follow-up:** pregnant women who have attended antenatal clinics during their recent pregnancy and have attended ANC at least once.

**Adequate knowledge of obstetric danger signs:** a delivered mother who could be able to mention obstetric danger signs that occur during pregnancy, childbirth, and the postpartum period above the calculated mean could be considered to have adequate knowledge, and those who could state below the mean were considered to have inadequate knowledge (14).

**TBAs:** a birth attendant who initially acquired the ability by delivering babies herself (9).

**Access to health service**s: mothers have access to health services if the distance to a health institution takes less than 1 h.

**Good waiting time to get ANC:** for pregnant women who have attended antenatal care waiting time is less than one hour during the visit (15).

### Data collection and data quality control

To assure quality, the data were collected through face-to-face interviews by eight trained midwives after 2-day data collection training was given to them together with two senior public health officer supervisors. The questionnaire for the quantitative study was structured and adapted from related literatures (9, 14, 15). The questionnaire was also prepared in English and translated to the local (Amharic) language and then translated back to English. A pretest was conducted on 5% of the respondents in the adjacent district, Bure to ensure the consistency of the questionnaire, and then the questionnaire was modified accordingly., but the results of the pretest were not included in the final analysis. For the qualitative study, focus group discussions and interviews were used to collect data. The data collection checklist contained open-ended questions and was adapted from previous similar studies (9, 16). The FGD and IDIs were intended to explore experiences, feelings, attitudes, thoughts, and ideas of discussants on determinants of home delivery.

### Data processing and analysis

Epi Info version 7 software was used for data entry, and SPSS version 24 was used for analysis. Bivariate logistic regression was employed to identify an association between independent and dependent (home delivery; Yes = 1, No = 0) variables. Variables with a *P*-value ≤ 0.25 in the bivariate logistic regression analysis were fitted into the multivariable logistic regression model. The 95% confidence interval of the odds ratio was computed, and a variable with a *P*-value < 0.05 in the multivariable logistic regression analysis was considered statistically significant.

**Qualitative:** In addition to recording in an audio file, field notes were taken for each interview. The audio file, consent form, field note, and in-depth enrollment form were coded properly and consistently. Every day, every recorded audio file was checked for its appropriateness in terms of proper coding, signed consent, clear audibility, level of depth questioning, and consistency of the codes given for the audio file, consent, and field note. Finally, the data were analyzed under selected themes and summarized manually.

### Ethical considerations

Ethical clearance for the study was obtained from the Research Institutional Research Ethics Review Committee of Debre Markos University (IRERC). Written and verbal informed consent was obtained from participants to confirm their willingness. Participants were interviewed in a separate room, and the anonymity and confidentiality of the data providers were strictly maintained.

## Results

### Quantitative study

#### Socio-demographic characteristics of the respondents

About 459 respondents participated in this study, with a response rate of 97.2%. The mean age of the respondents was 28 years. All respondents were Orthodox (459/100%), similar to all of the respondents' ethnicities. About 447 (97.3%) were married (Table 1).

**Table 1:** Socio-demographic characteristics of women who had home delivery after ANC follow-up in Jabitehnan District Northwest Ethiopia, 2019.

Variables	Frequency	Percent
Age		
<=24 years	119	25.9
25–34 years	272	59.3
>=35 years	68	14.8
Religion		
Orthodox	459	100
Marital status		
Married	447	97.4
Divorced	12	2.6
Occupation		
Farmer	240	52.3
Merchant	93	20.3
Daily laborer	126	27.5
Maternal education		
Unable to read and write	266	58.0
Able to read and write	169	36.8
Primary and above	24	5.2
Husband education		
Unable to read and write	177	38.6
Able to read and write	169	36.8
Primary and above	113	24.6
Ethnicity		
Amhara	459	100
Monthly income in ETB		
<2,000	143	31.2
2,001–4,000	209	45.5
>4,000	107	23.3

#### Obstetric and gynecologic-related characteristics of respondents

About 56% of the respondents were below gravida four, and 40.1% had two to four children. About 13.5% of the respondents attended ANC only once, and 61.4% of the respondents had completed ANC follow-ups. Only 192 (41.8%) of the women were decision-makers (Table 2).

**Table 2:** Obstetric and other health-related characteristics of women who had home delivery after ANC in Jabitehnan District, Norwest Ethiopia, 2019.

Variables	Frequency	Percent
Number of pregnancy		
one time	73	15.9
Two to four times	184	40.1
Five times and above	202	44.0
Number of delivery		
One time	47	10.2
Two to four times	210	45.8
Five times and above	202	44.0
Experience of abortion		
Yes	65	14.2
No	394	85.8
Previous place of delivery		
Health facility	199	43.4
Home	172	37.5
Place of ANC attend		
Health post	112	24.4
Health center	160	34.9
public hospital	187	40.7
The number of ANC attended		
First ANC	62	13.5
Second ANC	48	10.5
Third ANC	67	14.6
Fourth ANC	282	61.4
Pregnancy plan		
Planned	295	64.3
Unplanned	164	35.7
The final decision on the place of delivery		
Her self	192	41.8
Husband	39	8.5
Both my husband	166	36.2
parents	62	13.5
Distance from home to health facility		
<1 h	299	65.1
>=1 h	160	34.9
Any advice on where to deliver during the ANC		
Yes	302	65.8
No	157	34.2
Have information on free ambulance service		
Yes	455	99.1
No	4	0.9
Discussion habits with the husband on the place of delivery		
Yes	336	73.2
No	123	26.8
Visited by healthcare providers		
Yes	122	26.6
No	337	73.4

#### Knowledge of the respondents

Around 26.6% of study participants had adequate knowledge about the overall key danger signs of obstetric complications that can occur during pregnancy, labor, delivery, and the postpartum period (Table 3).

**Table 3:** Knowledge of key obstetric danger signs of home delivery after ANC and associated factors in Jabitehnan District Northwest Ethiopia, 2019.

Key danger signs during pregnancy	Frequency	Percent
Vaginal bleeding	203	44.2
Blurring of vision	86	18.7
Swelling of leg/face	87	19
Did not mention any danger sign	140	30.5
During labor and delivery		
Severe vaginal bleeding	161	35.1
Prolonged labor/>12 h	86	18.7
Retained placenta	37	8.1
Convulsions	12	2.6
Did not mention any danger sign	149	32.5
During postpartum		
Severe vaginal bleeding	300	65.4
Higher fever	16	3.5
Foul-smelling vaginal discharge	19	4.1
Convulsion	33	7.2
Did not mention any danger sign	110	24.0

#### Home delivery

In this study, the magnitude of home delivery after ANC follow-up among mothers who gave birth in the last 6 months was 37%, with a 95% CI of 32.5 and 41.5.

#### Factors associated with home delivery

After controlling the effect of other variables with logistic regression analysis, pregnancy plan [AOR: 4.562 (2.646, 7.864)], experience of abortion [AOR: 3.008 (1.631, 5.545)], place of ANC attendance [AOR: 1.886 (1.119, 3.179)], and visited by health care providers during ANC follow-up absence [AOR: 1.609 (1.023, 2.531)] were predictors of home delivery. Poor counseling during ANC, lack of planning, traditions, and beliefs were reasons for home delivery (Table 4).

**Table 4:** Multivariable logistic regression analysis of home delivery and associated factors in Jabitehnan District, Northwest Ethiopia, 2019.

Variable		Home delivery		COR	AOR
		Yes	No		
Maternal occupation	Farmer	76	164	0.58 (0.37, 0.90)	0.67 (0.42, 1.08)
	Merchant	38	55	0.86 (0.50, 1.49)	1.56 (0.83, 2.93)
	Daily laborer	56	70	1	1
Pregnancy plan	Planned	130	165	2.44 (1.60, 3.73)	**4.56** (**2.65, 7.86)**
	Unplanned	40	124	1	1
Experience of abortion	Yes	35	30	2.24 (1.32, 3.80)	**3.01** (**1.63, 5.55)**
	No	135	259	1	1
Place of ANC attended	Health post	35	77	1	1
	Health center	47	113	0.92 (0.54, 1.55)	1.10 (0.63, 1.91)
	Public hospital	88	99	1.96 (1.20, 3.20)	**1.89** (**1.12, 3.18)**
Visited by healthcare providers	Yes	56	66	1.66 (1.09, 2.53)	**1.61** (**1.02, 2.53)**
	No	114	223	**1**	**1**

COR, crude odds ratio; AOR, adjusted odds ratio. The bold indicates *P* value less than 0.05.

#### Qualitative study

### Socio-demographic characteristics

About 30 study respondents participated in this study. From these respondents, 19 participants were between the ages of 20 and 29 years old; 17 participants were unable to read or write; and 27 participants were in paraII and above.

#### Simple and sudden onset of labor

Pregnant women who had an ANC follow-up had full information on their expected date of delivery.

One woman explained: “*I presumed I had 10 days more, but I was mistaken. Consequently, labor started unexpectedly, so I gave birth at home with the help of my grandmother. I was not aware of my expected date of delivery. But I planned to give birth in the health facility with the help of health providers*” *(Participant # 9, age 26, and Para ii)*.

### Infrastructure and accessible transport

Accessibility to health facilities is a crucial issue in choosing health facility delivery. A 32-year-old woman asserted, “*Getting birth is an expected event and difficult to plan. It was midnight and raining when my labor started. My husband and relatives searched for a vehicle to take me to the health facility. But they could not get it yet. Walking on foot was not possible. Therefore, my last option was to call neighbors*’ *old women to help me and give birth at home*” *(Participant # 12, Para i)*.

Similarly, a 28-year-old woman who gave birth at home clearly stated that “*it is good to give birth at the health center. But the distance from my home to this health center is far, and the ambulance service is not easily accessible. Therefore, unless labor becomes difficult, it is better to give birth at home*” *(Participant # 13, Para ii).*

### Counseling during antenatal care follow-up

ANC follow-up is a golden time for health providers to enhance health facility delivery. But a 24-year-old mother said, “*I got birth at home. No one offered me a piece of advice on the importance of giving birth in a health center rather than giving vaccinations, and some tablets attended ANC three times regularly. Going through, I did not remember health care providers counseling me on the place of delivery*” *(Participant # 28, para v).*

A 34-year-old woman explained regarding time: “*Why do I spend my time visiting the health facility? Healthcare providers did not tell me something different. The follow-up was the same. I believe it is not as important for a healthy mother unless she gets sick*” *(Participant # 21, para ii)*.

Normal pregnancy during antenatal care follow-up might be considered an indication of normal delivery. One woman asserted that “*heath providers can predict any future birth complications. Therefore, if the provider told me my pregnancy was good, what is the importance of birth in a health facility? I understood the facility delivery required when the provider told me that my pregnancy is not normal*” *(Participant # 7, para iv)*.

Previous experience regarding health care provision motivated me to give birth at home. One 42-year-old woman stated, “*I have delivered my previous child at the health center. It was at night, and the health worker with untidy hair rising made me on the delivery coach; he left me alone for about 15 min and slapped and insulted me during delivery. Therefore, if the practice is like this, why should I go to the health center?*”

### Traditions and beliefs

The existence of psychological and social gaps between the community and healthcare providers may be a barrier to seeking facility delivery. Seven women said, “*Giving birth is not a disease; it is a natural life. Therefore, facility delivery is only for those unable to deliver normally and those who had the experience of stillbirth and spontaneous abortion previously*” *(Participant # 16, 12, 19, 3, 6, 11, 5)*.

Many participants believed traditional birth attendants were comfortable caregivers. Some women said that “*I gave birth by traditional birth attendant without complications or difficulty of labor*” *(participant #s 1, 4, 18, 23, 26, 29, 27, 30, 21). S*ome explained the issue of privacy based on their experience and said, “*Health providers are many in number at the delivery room; they took away privacy and performed several physical vaginal examinations, which were uncomfortable; therefore, how can I go to a health facility knowing what my elder daughter has experienced?*” *(Participant # 8, 17, 22, 24, 6, 2)*.

## Discussion

The magnitude of home delivery in this study was 37% [95% CI: 32.5%, 41.5%] which was in line with a study conducted in Adigrat (35%) (17), Arbaminch (39.9%) (18), and Nepal district (33%) (19).

In this study, home delivery is lower than in the 2016 Ethiopian demographic health survey (74%) (7) and the study was done among women who booked ANC in the Gozamin district (75.3%), Fogera district (68.4%), and Dodota (81.8%), in Ethiopia (7, 9, 11, 20). This difference might be due to time, which could be the government and other concerned bodies investing in different interventions to reverse the problem and study method, the data collection tools, and huge geographical coverage, particularly the Ethiopian demographic health survey. This study was also lower than the study conducted in Nyandarua, South District, Kenya (52%) (21). This could be due to different tribes with different cultural beliefs.

On the other hand, the magnitude of home delivery is higher than the findings of a study conducted in Bahir Dar town (21.2%), and Debre Markos town (19.5% and 25.3%) (15, 22, 23). This difference could be due to the residents having better infrastructure, accessibility, and education since the current study area is the rural community of Jabitehnan district. Similarly, the study was conducted in Tanzania (21.4%) (24). This might be due to the difference in health care services and the differences in culture. The previous study was conducted in the urban community of Dodoma region which might create media exposure, adequate health information, and better accessibility.

In the current study women who had unplanned pregnancies were 4.56 times more likely to have a home birth as compared with women who had planned pregnancies. This study was supported by studies done in Debre Markos town (15, 25). This might be due to the plan to have a child who particularly needs psychological and mental readiness. Again, planned pregnancy indicates the presence of a support person, either the husband or partner, which means having a chance to share ideas. Consequently, if pregnancy is planned and supported, the chance of seeking facility delivery increases. This is also supported by those women who were unaware of the expected date of delivery and explained *(Participant # 9, aged 26, para ii)*.

In the current study, the type of health institution for antenatal care follow-up was also found to be another predictor of home delivery. Attending ANC at a health post was 1.89 times more likely to give birth at home than those women who were attending ANC at a public health hospital. This is because in the hospital, most of the time there are midlevel and senior educated and experienced health professionals that enable them to receive proper counseling during ANC at the place of delivery, while in health care, a lack of such professionals and investigations led to an increase in home delivery (15).

Women who had no abortion experience were 3.01 times more likely to have home deliveries as compared to those who had no abortion history. The possible reason might be that learning from exposure might lead them to seek facility delivery, so those who had not experienced a problem previously may have high home delivery. This is supported by one's belief, which is learned from exposure to a problem *(Participant # 16, 12, 19, 3, 6, 11, 5).*

Another associated factor for this study was that visits by health care providers during ANC follow-up absence were 1.61 times more likely to give birth at home as compared with their counterparts. The possible explanation could be that healthcare providers might not arrive at a consensus regarding the importance of the service and the complications of home delivery. This idea was supported by one study participant *(participant # 21, Para ii)*.

Recall bias was the limitation of the study since the respondents were requested to respond about the events that happened 6 months prior to this study; high numbers of those unable to read and write may affect the study outcome.

This study's strengths included its community-based design, which improved the findings' generalizability by addressing mothers who were unable to visit healthcare facilities, and its mixed-methods data, which can address research questions that cannot be satisfactorily answered by using only qualitative or quantitative data collection techniques.

## Conclusion and recommendations

The study reveals a high magnitude of home delivery despite national expectations. Factors include pregnancy plans, health institution type, abortion experience, and absence from antenatal care. Poor counseling during ANC, lack of planning, traditions, and beliefs were reasons for home delivery. The government should increase awareness of the importance of planned pregnancy. Other stakeholders should create training in client-provider communication and counseling skills for health professionals. Healthcare professionals should take the opportunity to motivate mothers to attend facility delivery services during ANC follow-up.

## Data Availability

The raw data supporting the conclusions of this article will be made available by the authors, without undue reservation.
